# Left gastric vein to adrenal vein anastomosis: intraoperative solution for gastric venous congestion following extended distal pancreatectomy

**DOI:** 10.1093/jscr/rjae541

**Published:** 2024-08-28

**Authors:** Ramiro Manuel Fernández-Placencia, Carlos Luque-Vásquez Vásquez, José Belón-Supo, Eloy Ruiz, Francisco Berrospi, Juan Celis-Zapata

**Affiliations:** Department of Abdominal Surgery, Instituto Nacional de Enfermedades Neoplásicas, 2520 E Angamos Ave. Of. 216. Surquillo, Lima 15038, Peru; Department of Surgical Clinics, Universidad Peruana Cayetano Heredia, 430 Honorio Delgado Ave. San Martín de Porres, Lima 15102, Peru; Department of Abdominal Surgery, Instituto Nacional de Enfermedades Neoplásicas, 2520 E Angamos Ave. Of. 216. Surquillo, Lima 15038, Peru; Department of Abdominal Surgery, Instituto Nacional de Enfermedades Neoplásicas, 2520 E Angamos Ave. Of. 216. Surquillo, Lima 15038, Peru; Department of Abdominal Surgery, Instituto Nacional de Enfermedades Neoplásicas, 2520 E Angamos Ave. Of. 216. Surquillo, Lima 15038, Peru; Department of Abdominal Surgery, Instituto Nacional de Enfermedades Neoplásicas, 2520 E Angamos Ave. Of. 216. Surquillo, Lima 15038, Peru; Department of Surgical Clinics, Universidad Peruana Cayetano Heredia, 430 Honorio Delgado Ave. San Martín de Porres, Lima 15102, Peru; Department of Abdominal Surgery, Instituto Nacional de Enfermedades Neoplásicas, 2520 E Angamos Ave. Of. 216. Surquillo, Lima 15038, Peru

**Keywords:** extended distal pancreatectomy, gastric venous congestion, left gastric vein, venous reconstruction

## Abstract

Extended distal pancreatectomy often requires resection of vascular structures and adjacent organs, potentially leading to gastric venous congestion. This case report describes a 49-year-old female who underwent radical antegrade modular pancreatosplenectomy for pancreatic ductal adenocarcinoma. During the procedure, segmental gastric venous congestion was observed and resolved by anastomosing the left gastric vein to the left adrenal vein. The in-hospital postoperative recovery was initially uneventful; however, the patient was readmitted because of intra-abdominal fluid collection that was managed with antibiotics. Pathological examination confirmed moderately differentiated ductal adenocarcinoma with lymphovascular invasion. The patient received adjuvant mFOLFIRINOX therapy and remains disease-free 12 months after surgery with adequate patency of the anastomosis. This case highlights the importance of recognizing and addressing gastric venous congestion during radical antegrade modular pancreatosplenectomy to prevent complications, such as delayed gastric emptying or gastric necrosis, and proposes left gastric vein to left adrenal vein anastomosis as an effective intraoperative solution.

## Introduction

Distal pancreatectomy (DP) may require the resection of important vascular structures or adjacent organs when infiltration is present. Gastric venous congestion (GVC) has been reported after total pancreatectomy (TP) [1] and pancreaticoduodenectomy (PD) [2]. Unlike these procedures, DP usually preserves the left gastric vein (LGV). However, an extended procedure, such as radical antegrade modular pancreatosplenectomy (RAMPS), requires ligation of the LGV as part of the standard procedure [3], while preserving the right gastric vein (RGV) and right gastroepiploic veins (RGEV). To the best of our knowledge, this complication has not been reported following RAMPS.

Herein, we report a case where segmental GVC of the lesser curvature and posterior wall of the stomach was identified during RAMPS. This issue was resolved by anastomosing of the LGV with the left adrenal vein (LAV) to ensure adequate venous outflow.

## Case report

**Figure 1:**
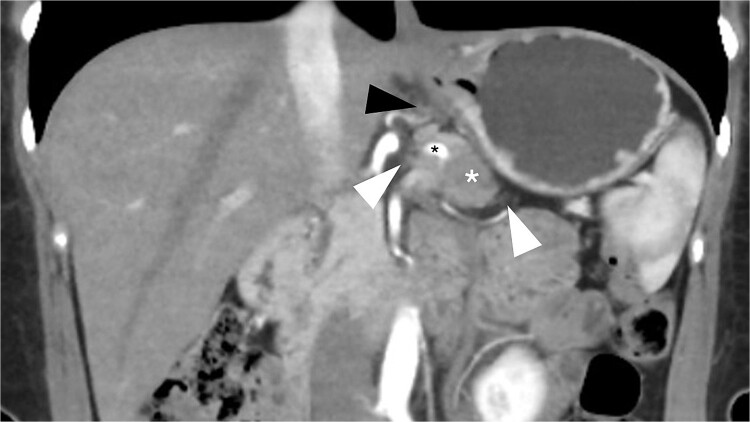
Preoperative CT scan (coronal view, portal phase) shows a 25 mm spiculated mass with hypoattenuation observed after the administration of IV contrast (white asterisk). The lesion encases the splenic artery (black asterisk), extends to the retroperitoneal fat (white arrowhead), and shows mesogastric distortion (black arrowhead).

A 49-year-old female patient underwent RAMPS for resectable pancreatic duct adenocarcinoma (PDAC) located in the body of the pancreas. Her preoperative CA19-9 levels were 20.51 IU/ml. Computed tomography (CT) revealed a 25 mm infiltrating mass at the level of the dorsal aspect of the body of the pancreas, associated with mild sinistral portal hypertension and invasion of the left adrenal gland, retroperitoneal fat, and splenic vessels (Fig. 1). Posterior RAMPS was performed to ensure adequate margins. During surgery, we confirmed that the mass had infiltrated the splenic vessels and LGV, and ingurgitation was observed along with other varicose veins in the stomach.

**Figure 2:**
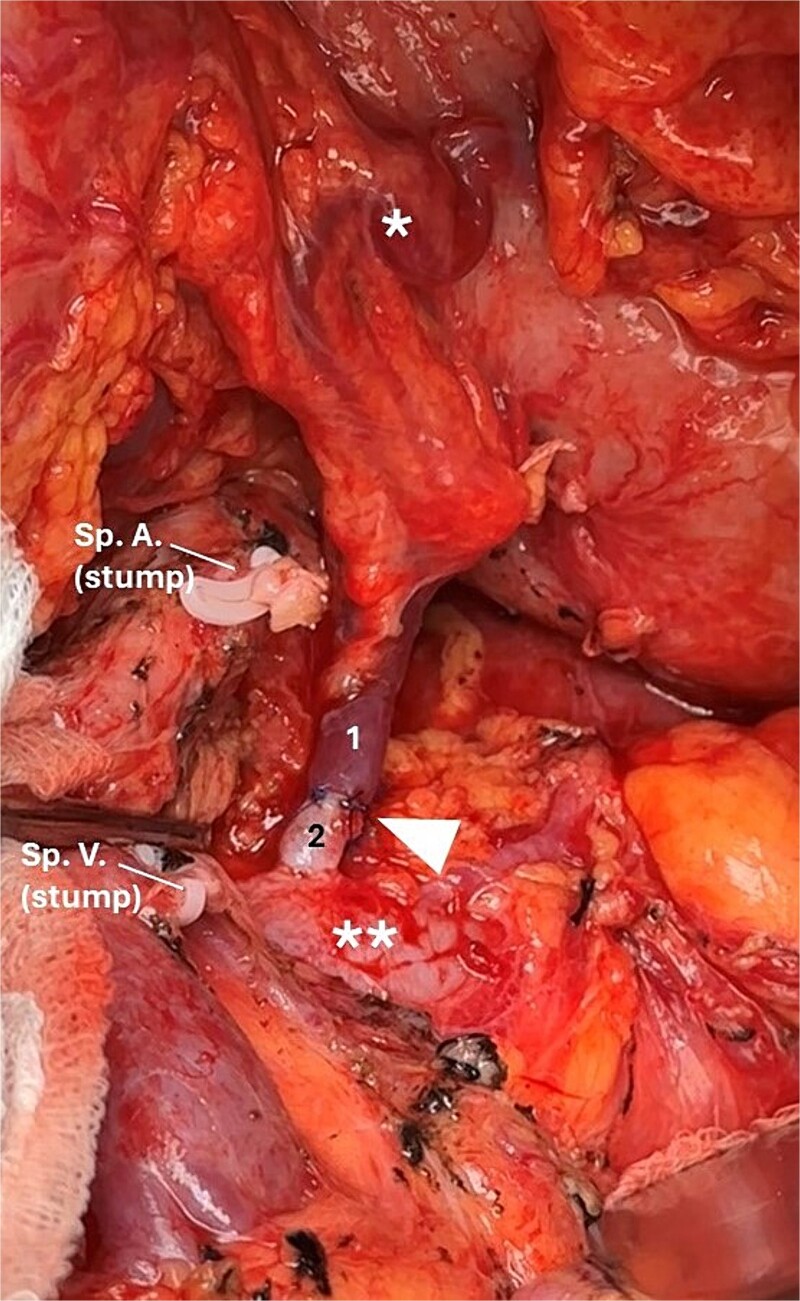
Final intraoperative view after the RAMPS procedure. Left gastric vein [1], left adrenal vein [2], and vascular anastomosis (white arrowhead). Variceal veins at the level of the lesser curvature (*). Left renal vein (**).

After excision of the tumor, persistent congestion of the lesser curvature and changes in the appearance of the posterior gastric wall, including petechiae and violet discoloration, were observed. To overcome this issue, adequate length of the LGV to reach the LAV to perform tension-free anastomosis was observed using a 6-0 polipropilene suture, which was approximated in a running fashion, and the anterior surface was performed using separate stitches. After unclamping, a significant reduction in congestion was observed; after 1 h, only the residual petechiae were observed. Previous varicose veins were found to have decreased in diameter (Fig. 2).

The patient’s postoperative recovery initially progressed without complications, allowing her to resume oral intake on the second day after surgery (POD2) and be discharged on the sixth day (POD6). However, she was readmitted on the tenth day post-operation (POD10) due to intra-abdominal fluid collection at the surgical site, which was managed with antibiotics. The patient was discharged on POD14. Throughout this period, there was no evidence of GVC or delayed gastric emptying (DGE), and the anastomosis remained patent.

**Figure 3:**
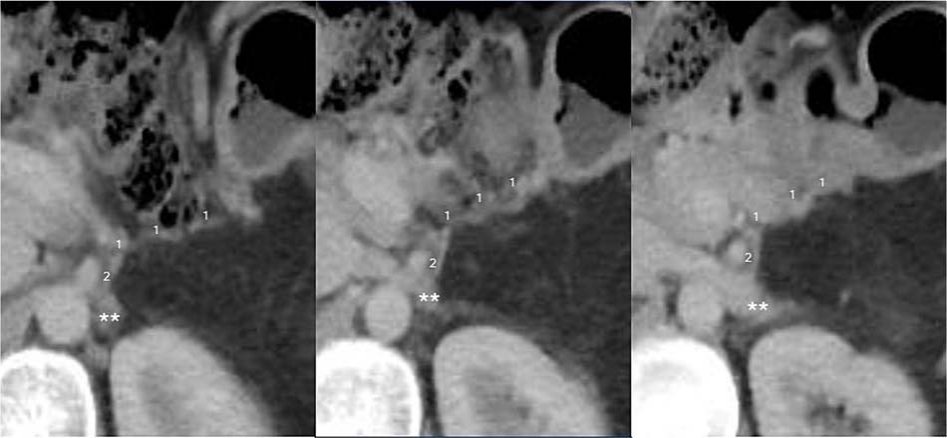
CT scan taken 12 months after surgery, displayed from upper to lower in a left to right sequence, showing the anastomosis between the left gastric vein (LGV, 1) and left adrenal vein (LAV, 2). The left renal vein is indicated by ‘^**^’.

The final pathological examination revealed a 38 mm moderately differentiated ductal adenocarcinoma (T2). The 5/12 lymph nodes (N2) and lymphovascular and perineural invasion were positive, and the surgical margins were negative for malignancy. Thereafter, the patient received adjuvant treatment with FOLFIRINOX with adequate tolerance. Twelve months after surgery, there was no indication of venous congestion or recurrence of the disease, and the venous anastomosis continued to exhibit good patency (Fig. 3). Additionally, her CA19-9 level remained stable at 19.82 IU/ml.

## Discussion

GVC is seldom reported as an adverse outcome of pancreatic surgery, although it may exist in 5%–28% of patients undergoing TP [4]. Herein, we present a case in which GVC resulted after RAMPS, which was mainly observed as a segmental discoloration of the gastric body and fundus. Therefore, we aimed to restore adequate outflow to prevent DGE or necrosis.

It has been previously reported that ligation of the LGV was associated with an increased risk of DGE [2, 5]. As demonstrated by Zhang *et al.* [6], the gastroepiploic vein may not always provide sufficient outflow to the stomach. In patients undergoing TP, LGV ligation is an independent risk factor for GVC, which may also be related to ligation of the splenic vein [4]. LGV is the primary route of venous outflow of the stomach after disconnection of the splenic vein from the mesentericoportal junction; when restoring adequate gastric outflow is obviated, venous ischemia or DGE may occur [7].

In our experience after RAMPS and TP, GVC has not been previously observed. Yet, in this particular case, despite adequate preservation of the RGV and RGEV, segmental GVC likely resulted from a combination of factors. These included the extensive tumor infiltration of the LGV and splenic vein with the resulting sinistral portal hypertension. Specifically, we observed segmental congestion affecting only the lesser curvature and posterior wall of the stomach, extending from the body to the fundus. Probably, in this patient, the normal patterns of venous drainage [8] were insufficient. Consequently, after ligating the LGEV and the LGV, congestion occurred and was promptly resolved following the vascular anastomosis.

This case report provides an alternative for reconstructing the outflow of patients with GVC after RAMPS. If deemed necessary, it can provide an alternative reconstruction after TP.
